# Strategies to improve atrioventricular synchrony in patients with a Micra AV leadless pacemaker

**DOI:** 10.1093/europace/euae060

**Published:** 2024-03-07

**Authors:** Christophe Garweg, Alexander Breitenstein, Nicolas Clémenty, Carlo De Asmundis, Saverio Iacopino, Jens Brock Johansen, David Sharman, Cathrin Theis, Xavier Viñolas Prat, Stefan Winter, Tobias Reichlin

**Affiliations:** Department of Cardiovascular Sciences, University Hospitals Leuven, Herestraat 49, 3000 Leuven, Belgium; Center for Cardiac Arrhythmias and Cardiac Electrophysiology, UniversitätsSpital Zürich, Zurich, Switzerland; Cardiology Department, Clinique du Millenaire, Montpellier, France; Heart Rhythm Management Center, University Hospital Brussels, Brussels, Belgium; Department of Arrhythmia and Electrophysiology, Maria Cecilia Hospital, Cotignola, Italy; Department of Cardiology, Odense University Hospital, Odense, Denmark; Cardiology Service, Northampton General Hospital NHS Trust, Cliftonville, UK; Department of Cardiology, Robert-Bosch-Krankenhaus Stuttgart, Stuttgart, Germany; Department of Cardiology, Sant Pau Hospital, Barcelona, Spain; Cardiology and Rhythmology, Saint Vinzenz Hospital, Cologne, Germany; Department of Cardiology, Inselspital—University of Bern, Bern, Switzerland

**Keywords:** Leadless pacemaker, AV synchrony, VDD pacemaker, Patient selection

## Abstract

The second generation of transcatheter pacing systems, called Micra AV, can provide atrioventricular (AV) synchronous pacing via a new pacing algorithm relying on sensing mechanical atrial contraction. Several novel programming parameters were introduced to enable AV synchronous pacing, including an A3 window and A4 window as well as a conduction mode switch and an activity mode switch. In addition to several automated features, manual programming optimization of some of the novel parameters is key to improving AV synchrony. A solid knowledge of the features and their programming is essential for electrophysiologists implanting or following patients with Micra AV devices. Differences in programming optimization might partially explain the high variability of AV synchrony published in real-world data reports. This article reviews the key programming parameters of Micra AV. Subsequently, optimal programming recommendations for defined patient profiles are presented. Those were established by consensus within an expert panel comprised of 11 European electrophysiologists from high-volume Micra AV centres. The patient profiles were (1) high degree AV block and slow sinus rhythm; (2) high degree AV block and fast sinus rhythm; and (3) intermittent AV block. The panel recommended to evaluate the mechanical atrial activity on transthoracic echocardiography prior to implant. It was also agreed that Auto A3 Threshold and Tracking Check should be turned off in all patients, AV conduction mode switch should be turned off in all patients with high degree AV block, and the lower rate should be programmed to 50 bpm with exceptions based on individual clinical assessment. Future studies will be useful to evaluate the strength of those recommendations to improve the AV synchrony.

## Graphical Abstract

**Graphical Abstract:**
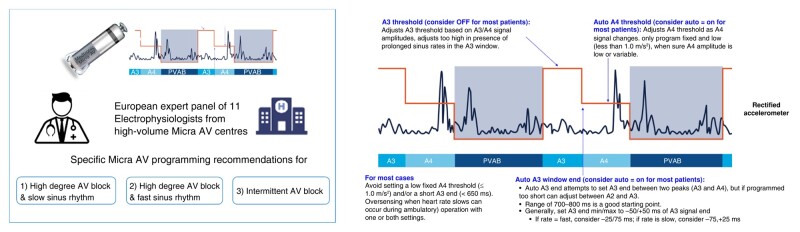

**What’s new?**

- Recently published real-word data showed that a Micra AV leadless pacemaker implanted in the right ventricle can provide atrioventricular (AV) synchronous pacing by accelerometer-based mechanical sensing of atrial contractions in patients with a high ventricular pacing burden.
- An expert panel, made up of 11 European electrophysiologists from high-volume Micra AV centres, agreed on general recommendations for Micra AV patient management aimed at improving AV synchrony.
- The panel recommended to evaluate the mechanical atrial activity on transthoracic echocardiography prior to implant.
- It was also agreed that Auto A3 Threshold and Tracking Check should be turned off in all patients, AV conduction mode switch should be turned off in all patients with high degree AV block, and the lower rate should be programmed to 50 bpm with exceptions based on individual clinical assessment.

## Introduction

Since their clinical introduction in 2013, leadless pacemakers have had a growing place in the treatment of bradyarrhythmia requiring the implantation of a pacemaker. The first generation of leadless transcatheter pacing systems (Micra™ VR, Medtronic Inc., MN, USA) has demonstrated strong safety and efficacy performance reducing the risk of major complications by up to 63% at one year follow-up in comparison with a historical group of patients and with a 31% reduction of complications at 2 years follow-up in the Micra Coverage with Evidence Development (CED) Medicare claims study.^1,2^ However, Micra VR delivers only ventricular pacing and sensing (VVIR mode), limiting its use to patients with permanent atrial fibrillation and bradycardia or to patients having a precluding condition for the use of transvenous pacing systems.

To benefit from the recognized advantages of atrioventricular (AV) synchrony, a second generation of leadless pacemakers, the Micra AV (MC1AVR1, Medtronic Inc., MN, USA), was developed to enable an atrial mechanical sensing VDD pacing mode while still using a single device implanted in the right ventricle. The MARVEL 2 study (Micra Atrial tRacking using a Ventricular accELerometer) reported an 89.2% mean rate of AV synchrony at rest in patients with high degree AV block and normal sinus rhythm.^3^ Subsequent publications reflective of the real-world experience with Micra AV reported ambulatory AV synchrony rates ranging from 33–91%, with variation noted based upon heart rate, pacing indication, and percentage of ventricular pacing.^4–8^ Notably, a sub-analysis of the AccelAV study, AccelAV-Optimize demonstrated the efficacy of prescribed programming changes on ambulatory AV synchrony, with an average AV synchrony gain of 10% from 71.9–82.6%.^6^ Routinely performed programming optimization of several novel parameters (particularly concerning the A3 and A4 windows) appears to be an important key to improving AV synchrony.

Recent international guidelines and EHRA position papers have clarified the current clinical indications of leadless pacing systems, but at present, there is no general consensus on the optimal programming strategy of Micra AV devices.^9,10^ The aim of this project is to propose an expert consensus statement, based on available literature, experts’ opinions and experiences on how to adequately program the Micra AV leadless pacemaker.

## Methods

### Panel composition and panel meetings

The 11 members of the panel represented implanters of leadless pacing systems from high-volume centres (37 ± 17 Micra AV procedures/year) in Europe contributing to the prospective Micra AV Post-Approval Registry (PSR). The selection criteria were: affinity and knowledge of the Micra AV transcatheter pacing system, experience with Micra AV (at least 20 previous implant procedures), the willingness to invest time to participate in the project (∼2–4 h of preparation), and four online face-to-face panel meetings.

The experts shared their opinion about the optimal programming strategy to improve AV synchrony in three different typical patient profiles: (1) patients with slow sinus rhythm and high degree AV block, (2) patients with fast sinus rhythm and high degree AV block, and (3) patients with intermittent AV block. Programming strategies discussed were applicable to the Micra AV device (Model MC1AVR1).

To reach a consensus, the following steps were followed:

- An online survey was completed by the panel to evaluate their current programming strategy, based on PSR data.
- After a face-to-face meeting to discuss the survey results, a second survey round was completed to reach consensus on the optimal programming strategy. Agreement was considered reached when >75% of responders agreed.
- A final survey round after one year was performed to collect the physicians’ feedback on the feasibility of the optimized programming strategy and additional recommendations after one year of experience.
- Although the project was funded by Medtronic, the company did not influence the proposed programming strategy by the panel experts.

### Micra AV: accelerometer signals and specific ‘pacing’ windows

**Figure 1:**
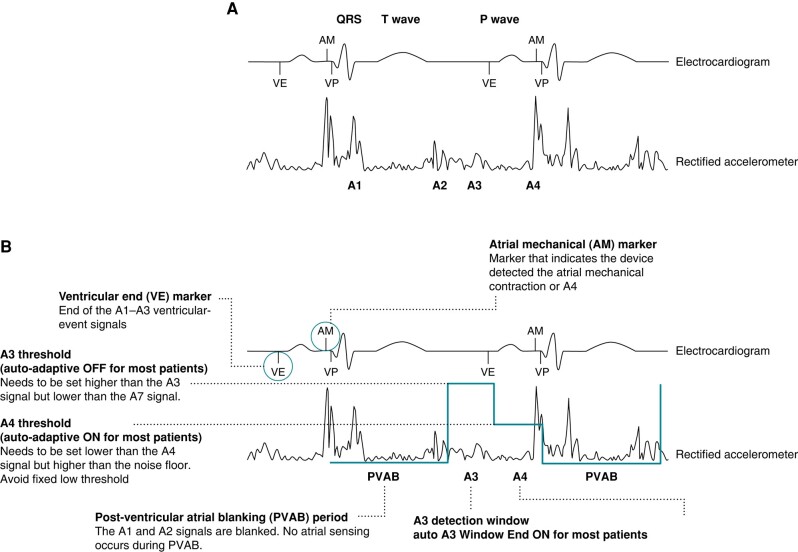
(*A*) The four different accelerometer signals. (*B*) Specific Micra AV parameters and adapted programming parameters.

The Micra AV device accelerometer measures mechanical activity during the cardiac cycle and uses this information to deliver AV synchronous ventricular pacing. A single cycle’s activity can be characterized into four signals (A1–A4), the latter two of which are measured by the device to enable AV synchronous ventricular pacing (*Figure 1A*).

- A1 signal: occurs at the beginning of ventricular systole and represents the closing of the mitral and tricuspid valves.
- A2 signal: occurs at the completion of ventricular systole and represents the closing of the aortic and pulmonary valves.
- A3 signal: occurs during ventricular diastole, corresponds in timing to the E-wave of the mitral inflow on a Doppler echocardiogram, and represents the passive filling of blood from the atrium into the ventricle.
- A4 signal: occurs when the atrium contracts and pushes blood into the ventricle and corresponds in timing to the A-wave of the mitral inflow on a Doppler echocardiogram.

## Overview of the novel Micra AV markers and programming parameters

The novel Micra AV markers and programmable parameters are summarized in *Figure 1B*. Specifically, in addition to the VP (ventricular pace) or VS (ventricular sense) marker on the device’s electrocardiogram, the device provides a VE marker and AM marker. The VE marker stands for ‘Ventricular End’, and it indicates the end of the ventricular events (end of A1–A3 signals) and is determined by the end of the A3 window. The AM marker indicates the device detected an atrial mechanical contraction (A4) on the accelerometer. AM detection sensitivity is determined by the post-ventricular atrial blanking (PVAB), post-ventricular atrial refractory period (PVARP), A3 threshold, A3 detection window, and A4 threshold that are all programmable, as described in *Table 1*.

**Table 1:** Programming recommendations by patient profile

	Patient profile		
Programmable parameter	Slow sinus rhythm and high degree AV block	Fast sinus rhythm and high degree AV block	Intermittent AV block
Auto A3 Window End	ON A3 Window End range of 700–800 ms as a good starting point. Set A3 Window End Min/Max to −50/+50 ms of A3 signal end. If rate = fast, consider −25/75 ms; if rate is slow, consider −75,+25 ms.		
Auto A3 Threshold	OFF Adjusts A3 Threshold based on A3/A4 signal amplitudes, adjusts too high in presence of prolonged periods of high sinus rates in the A3 window. Program the A3 Threshold to a fixed value 1.0–1.5 m/s^2^ greater than an isolated A3 signal.		
Auto A4 Threshold	ON Adjusts A4 Threshold as A4 signal changes. Only program fixed and low (<1.0 m/s^2^), when sure A4 amplitude is low (<1.2 m/s^2^) or variable.		
PVAB/upper tracking	The nominal of 550 ms works for most patients. A shorter value can be programmed in patients with small or early A2 signals. If 500 ms is programmed, an upper tracking rate of 115 bpm can be programmed.		
Auto PVARP	The nominal max PVARP of 600 ms works for most patients. This parameter guards against A2 oversensing if A2 signal occurs later at slow rates.		
Rate smoothing	Nominal = 100 ms		
	It can be programmed longer if high sinus variability is observed at low rates.	Consider programming to 50 ms in patients with elevated sinus rates.	
AV conduction mode switch	Patient has AVB, no need to enable feature.		Program ON unless patient has idioventricular rhythm or 2:1 AVB with ventricular rates > 40 bpm.
Activity mode switch	ON It can provide rate support during patient activities that are not tracked by the device.		Consider programming OFF, if patient has intrinsic conduction and normal sinus function most of the time.
Tracking Check	OFF It may disrupt tracking at high sinus rates.		
Lower rate programming	50 bpm works for most patients. Measure sinus rate at rest.		
	If sinus rate < 60 bpm, consider programming lower rate to 45 or 40 bpm.	Sinus rate > 60 and <100 bpm	If sinus rate > 60 bpm
		And if sinus rate is not anticipated to drop below 60 bpm at night, a lower rate of 60 bpm can be programmed.	

## Expert panel recommendations for the management of patients with Micra AV devices

### I. General recommendations before and at the implant procedure (*Figure 2*).

**Figure 2:**
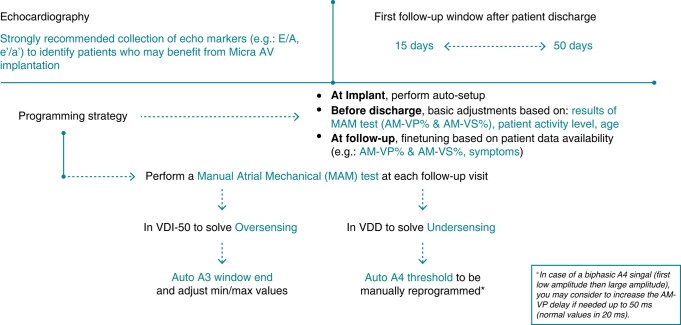
General recommendation for Micra AV patients’ management.

As the Micra AV algorithm relies on the mechanical atrial signal detection, a systematic approach starting at the implant procedure is imperative. Regarding patient selection, although the primary decision to implant a Micra AV device is based on a number of trade-offs with respect to patient risks and preference, it is recommended to perform a pre-implant echocardiogram with a particular focus on diastolic function and atrial function to identify patients who are most likely to achieve high levels of AV synchrony from a Micra AV implantation (*Figure 2*).^11^ In the presence of impaired atrial function (E/A > 1.5), the expected AV synchrony will be lower and it is recommended to evaluate the need for higher degrees of AV synchrony vs. the benefits provided by a single-device leadless pacemaker in each case.

The implant procedure itself is the same as for the Micra VR and has been previously described.^12,13^ At the end of the implant procedure, the automatic Micra AV autosetup (‘Atrial Sensing Setup’) should always be performed. Finally, before discharge, basic adjustments based on results of the Manual Atrial Mechanical (MAM) test, patients’ activity and profile (see below) should also be performed.

### II. General recommendations for patient follow-up (*Figure 2* and *Figure 3A* and *B*).

**Figure 3:**
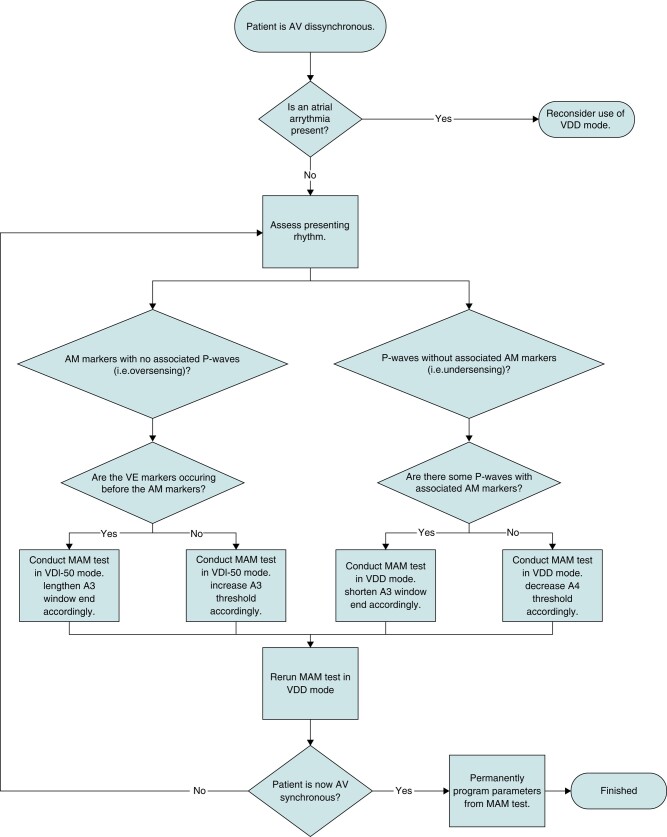

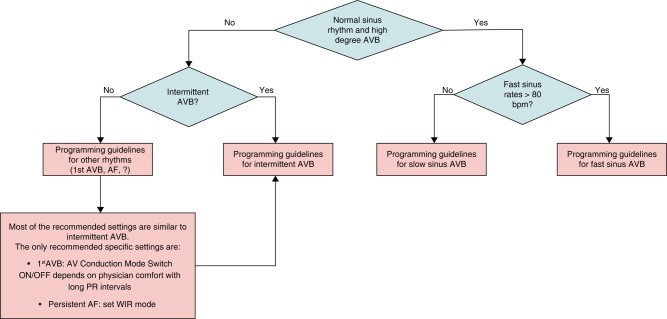
(*A*) Micra AV general programming recommendations. (*B*) Micra AV specific programming recommendation for different patients’ profiles.

A systematic approach for each follow-up is recommended. A first follow-up visit is recommended 15–50 days post-implant procedure to adjust the pacing parameters. Further patient follow-up should be performed as recommended by the ESC guidelines.^9^ Following the clinical assessment, different steps should be performed:

1. Continued acquisition of noiseless electrocardiogram lead for identification of the P wave.
2. Review of the device data on the percentage of the following sequences: AM-VP, (AM-VS, VS only, VP only, AV conduction mode switch (period with intrinsic ventricular rhythm > 40 bpm), and activity mode switch (time in VDIR mode) since the last interrogation session.
3. Analysis of the rate histogram and the %AM-VP and other counter values in AV histogram should be assessed across different heart rate bins. A physiological histogram generally indicates appropriate device function. A peak in the AV histogram with high AM-VP% (typically at the programmed A3 end interval) is usually indicative of atrial (A3) oversensing.^7^
4. Acquisition of a MAM test. This test permits evaluation of AV synchrony, sensing issues in office, and gives the opportunity to measure manually the A3 and A4 amplitudes. Performing the MAM test in VDI mode will aid in resolving oversensing issues while performing the test in VDD mode will help diagnose A4 undersensing. The A4 signal sometimes can be biphasic (initial low amplitude followed by high amplitude) requiring extension of the AM-VP delay from 20 ms up to 50 ms.
5. Exercise testing during routine follow-up is not necessary but is recommended when a patient presents with symptoms during exercise.

### III. Specific recommendations on programming parameters related to A3 and A4 signals: Auto Accelerometer Vector, A3 Threshold, Auto A3 Window End, and Auto A4 Threshold (*Table 1*, *Figures 1*, *3A*, *4*, and *5*)

**Figure 4:**
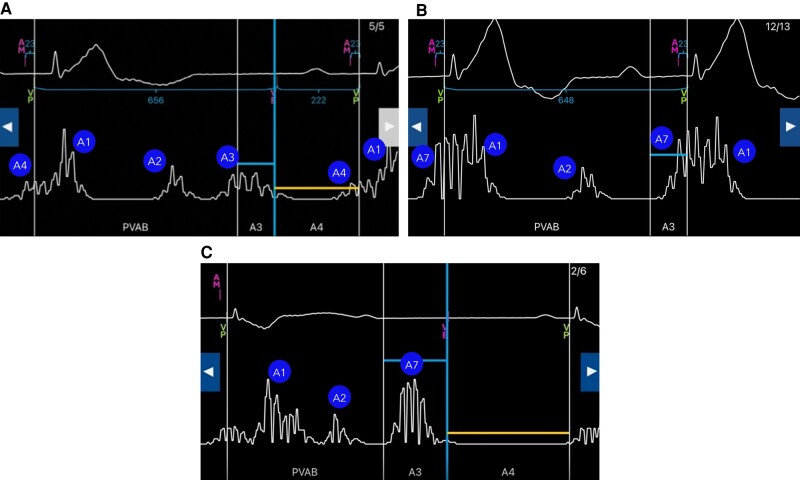
Auto A3 Threshold. The auto A3 threshold adjusts its maximal value on the maximal A3 amplitude sensed across the latest 24 h. (*A*) At lower rates, separate A3 and A4 signals can be well-identified. (*B*) At higher rates (typically >85/min), the A3 and A4 signals summate creating an A7 signal. (*C*) Example of the undersensing that can occur when the Auto A3 Threshold adjusts the A3 threshold too high.

**Figure 5:**
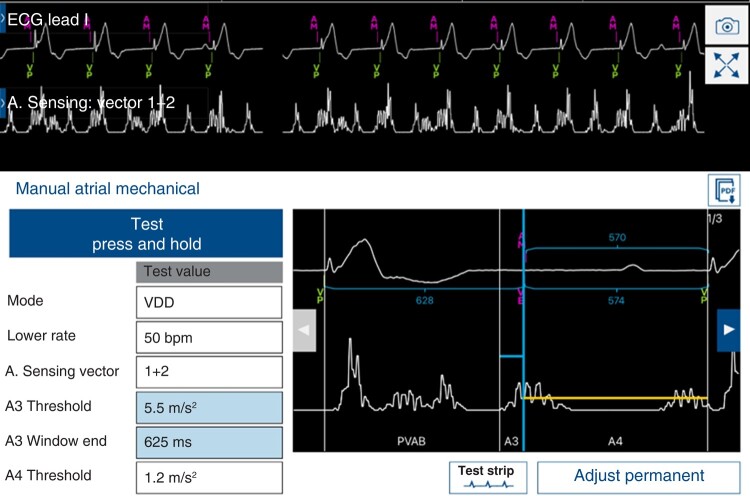
Auto A3 Window End. A Manual Atrial Mechanical test shows here an A3 window end that is set too short. This can result in oversensing the A3 signal in the A4 window.

Micra AV employs multiple accelerometer vectors and different accelerometer vector combinations can be chosen to increase the A4 amplitude. Micra AV employs a dual thresholding scheme. The A4 threshold is programmed to detect the A4 signal in isolation when the heart rate is slow and accelerometer signals due to passive filling (A3) and active filling (A4) are separated in time. The A3 threshold should be programmed higher than the isolated A3 signal, but low enough to detect the accelerometer signal at faster rates when the A3 and A4 signals fuse into a combined A7 signal.

1. Accelerometer vector—The autosetup algorithm generally chooses the 2 accelerometer vector combination with the highest A4 amplitude. If accelerometer signals are small in a patient, the quickest and simplest solution is to utilize all three vectors (1 + 2 + 3). This utilizes all vectors to maximize the A4 amplitude with only about a 2–3 month decrease in device longevity.
2. Auto A3 Threshold (*Figure 4*)—If Auto A3 Threshold is enabled, the device automatically adjusts the height of the A3 threshold on the basis of the automatic A3 and A4 measurements (see Supplementary material online, *Figure S1*). However, during prolonged periods of high sinus rates > 85 bpm, the A3 threshold increments higher than the A3 signal, causing undersensing of the A7 signal. If Auto A3 Threshold is disabled, the A3 Threshold operates at the fixed programmable value. To maintain AV synchrony at higher sinus rates, the recommendation of the expert panel is therefore to disable A3 threshold in most patients and adjust the A3 threshold based on measured A3/A4 signal amplitudes. The experts recommend setting the A3 threshold to a fixed value 1.0–1.5 m/s² higher than the measured A3 amplitude.
3. Auto A3 Window End (*Figure 5*)—The A3 Window End defines the boundary between the A3 and A4 thresholds. The A3 Window End is programmable over a wide range (650–1000 ms). The A3 Window End should be programmed at the end of the accelerometer A3 signal. The A3 Window End can be manually programmed by viewing an isolated A3 signal and programming the A3 Window End slightly longer than the A3 signal. The ‘Auto A3 Window End’ includes an automatic adjustment feature that will increase the A3 Window End when the sinus rate slows or A3 signal occurs later and will decrease the A3 Window End when the sinus rate increases or the A3 signal occurs earlier. If Auto A3 Window End is programmed to OFF, the A3 Window End operates at the fixed programmable value. According to the expert panel, this feature should be enabled and a range of 700–800 ms is a good starting point for most patients. The range of the window (A3 Window End Min/Max) can be generally set to −50/+50 ms of A3 signal end. If the patient has a relatively fast heart rate (>85 beats/min), −25/75 ms should be considered. For patients with a heart rate slower than 60 beats/min, values of −75/+25 ms should be considered. In general, a short A3 End (<650 ms) should be avoided since oversensing can occur when heart rates slow. There is still some debate in the literature about optimal programming of this feature in patients with relatively small A3 signals. In the AccelAV study, Minimum and Maximum A3 Window End mean values were 700 and 800 ms, respectively, independent of the A3/A4 signal measurements.^6^ In patients with small A3 signals, a recent study by Briongos-Figuero *et al*.^5^ has shown good AV synchrony with shorter A3 Window End values, closer to 650 ms. This strategy can work well in the presence of high A4 signals where the auto A4 threshold increases the A4 threshold well above the A3 signal. However, the experts estimate that there is a potential for oversensing the A3 signal when a short A3 Window End is used with a fixed and low A4 threshold.
4. Auto A4 Threshold—This feature automatically adjusts the height of the A4 threshold to detect the A4 signal with an adequate safety margin. If Auto A4 Threshold is enabled, the device will change the A4 threshold based on the signal in the A4 window and the number of A4 detections in recent history. If Auto A4 Threshold is disabled, the A4 Threshold operates at the fixed programmable value. If the A4 Threshold value is set too high, the device may undersense the atrial contraction, thus resulting in a loss of AV synchrony. The expert panel recommends enabling the auto A4 threshold to all allow the A4 threshold to change as the A4 amplitude changes. However, a fixed A4 threshold could be programmed for patients with low or variable A4 amplitude (<1.0 m/s^2^).

### IV. Specific recommendations on pacing parameters and modes switch (*Table 1*)

**Figure 6:**
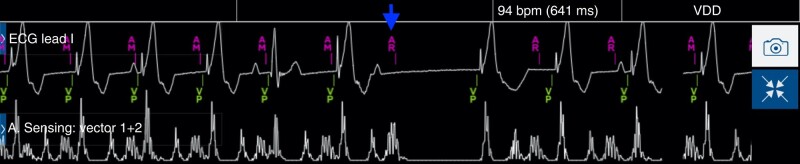
Illustration of the activated ‘tracking check’ feature at fast sinus rhythm. To guard against oversensing in the A3 window, tracking check extends PVARP for 1 cycle, causing the next cycle to fall within the refractory period (arrow).

1. AV conduction mode switch—Micra AV mode switches to VVI 40 (called VVI + mode) during periods of intact AV conduction to promote intrinsic rhythm in patients with episodic AV block. The expert panel suggests leaving this feature OFF for patients with permanent AV block and to turn it ON in patients with intermittent block. It is also important to keep in mind that while the VVI + mode is activated, the device remains in VVI mode in the presence of an intrinsic ventricular rhythm > 40 bpm regardless of the AV synchrony.
2. Activity mode switch—This feature, aiming to provide rate support during activity, switches to VVIR pacing if the sensor rate is at the Activities of Daily Living Rate and is 20 bpm higher than the VDD rate. The expert panel suggests enabling it since it can provide rate support during patient activities that are not tracked by the device. It should be disabled if the patient has intrinsic conduction and normal sinus function most of the time.
3. Tracking Check (*Figure 6*)—The tracking check feature was designed to guard against oversensing in the A3 window and to confirm the Micra AV is appropriately tracking the sinus rhythm when the device is tracking at or above the Tracking Check Rate (nominally 100 bpm but manually programmable at lower rate as 85 bpm). The feature operates by extending PVARP for 1 cycle, causing the next cycle to fall within the refractory period. The device estimates the location of the next AM detection and tracking is confirmed if the subsequent AM sense occurs at the expected time. If Tracking Check does not confirm appropriate tracking, PVARP will remain extended, limiting the high tracking rate. The tracking check feature is nominally ON in the Micra AV device, but during the AccelAV study, the specificity of the tracking check was not 100% due to variability in A4 detection timing.^6^ Therefore, the tracking check function intermittently failed even when the device was tracking appropriately. Conversely, oversensing in the A3 window was not observed during the study, calling into question the need for the feature and leading to the recommendation by the expert panel to turn the feature OFF.
4. Post-ventricular atrial blanking (PVAB)—This feature aims to blank the A2 signal. The expert panel suggests leaving the nominal value of 550 ms that works for most patients. Some patients have later A2 signals at low sinus rates. If AV synchronous tracking is desired above 105 bpm, the PVAB will need to be programmed <550 ms. However, this can be challenging in the event of large and late A2 signals. Therefore, the amplitude and timing should be assessed with a MAM test to ensure that the A2 is not falling in PVAB. If A2 will not be detected in the A3 window, a shorter value could be programmed in patients to achieve a higher upper tracking rate (see Supplementary material online, *Figure S2*).
5. Auto PVARP (post-ventricular atrial refractory period)—This feature adjusts PVARP lower than 600 ms as rate decreases and works in conjunction with PVAB to guard against oversensing of the A2 signal. Accelerometer signals that are detected in the A3 window, but occur within PVARP, will generate an AR marker, but not be tracked by the device. The nominal max PVARP of 600 ms is acceptable for most patients and guards against A2 oversensing if the A2 signal occurs later at slow rates. Like the PVAB parameter, the max PVARP can be shortened if A2 signals are early or small.
6. Sensed AV (AM-VP) interval—The nominal AM-VP is 20 ms and should remain at the nominal setting for most patients. The A4 signal measurement occurs during the A4 window. In some patients, the full peak of the A4 signal is not measured in the 20 ms interval between the A4 detect (AM) and the VP. This will lead to the algorithm measuring a lower A4 measurement and possible result in the auto A4 threshold not increasing appropriately. This can often be solved by slightly increasing the AM-VP from 20–40 ms, to include the full A4 signal in the A4 (AM-VP) window. This 20 ms lengthening of the AM-VP will only slightly reduce the upper tracking rate. Also, the AM-VP interval can be programmed to reduce ventricular pacing in patients with 1st degree AVB. An AM-VP of up to 100 ms can promote AV conduction in some of these patients. Programming an AM-VP of >100 ms further reduces the effective upper tracking rate and could allow for long AV intervals to occur, so we believe that this trade-off should be made with caution.
7. Rate smoothing—This feature aims to improve AV synchrony during intermittent A4 undersensing. The expert panel suggests programming to 50 ms (nominal value is 100 ms) in patients with elevated sinus rates. Rate smoothing can be programmed longer in patients with higher sinus variability.

The programming strategies provided by the expert panel are specific to Micra AV model MC1AVR1. An updated version of the device Micra AV2 (MC2AVR1)^14^ received FDA approval in April 2023 and CE Mark in January 2024; however, Micra AV2 was not discussed by the European expert panel. In the Micra AV2 device, Auto PVAB features allow for a dynamic PVAB that makes it possible to have a higher programmable upper tracking rate of 135 bpm. Additionally, Micra AV2 has a new Auto + A3 Threshold feature that more accurately adjusts the A3 Threshold, so the device setup no longer includes setting a fixed A3 Threshold after autosetup completes.

## Conclusions

Specific programming recommendations for patients with slow sinus rhythm and high degree AV block, fast sinus rhythm and high degree AV block, and intermittent AV block have been developed by a European expert panel and are presented. Future studies are recommended to evaluate its strength to improve AV synchrony.

## Supplementary Material

euae060_Supplementary_Data

## Data Availability

There were no data collected for the purposes of analysis in the course of this project.
