# Supplementary material for: Strategies to improve atrioventricular synchrony in patients with a Micra AV leadless pacemaker
Source: Europace. 2024 Mar 7;26(3):euae060. doi: 10.1093/europace/euae060 (PMC10936979; doi:10.1093/europace/euae060)
Supplement: euae060_Supplementary_Data [file euae060_supplementary_data.docx]

**Supplementary Appendix**

**Supplementary Figure 1. Auto-measurments of the A3 amplitude.**


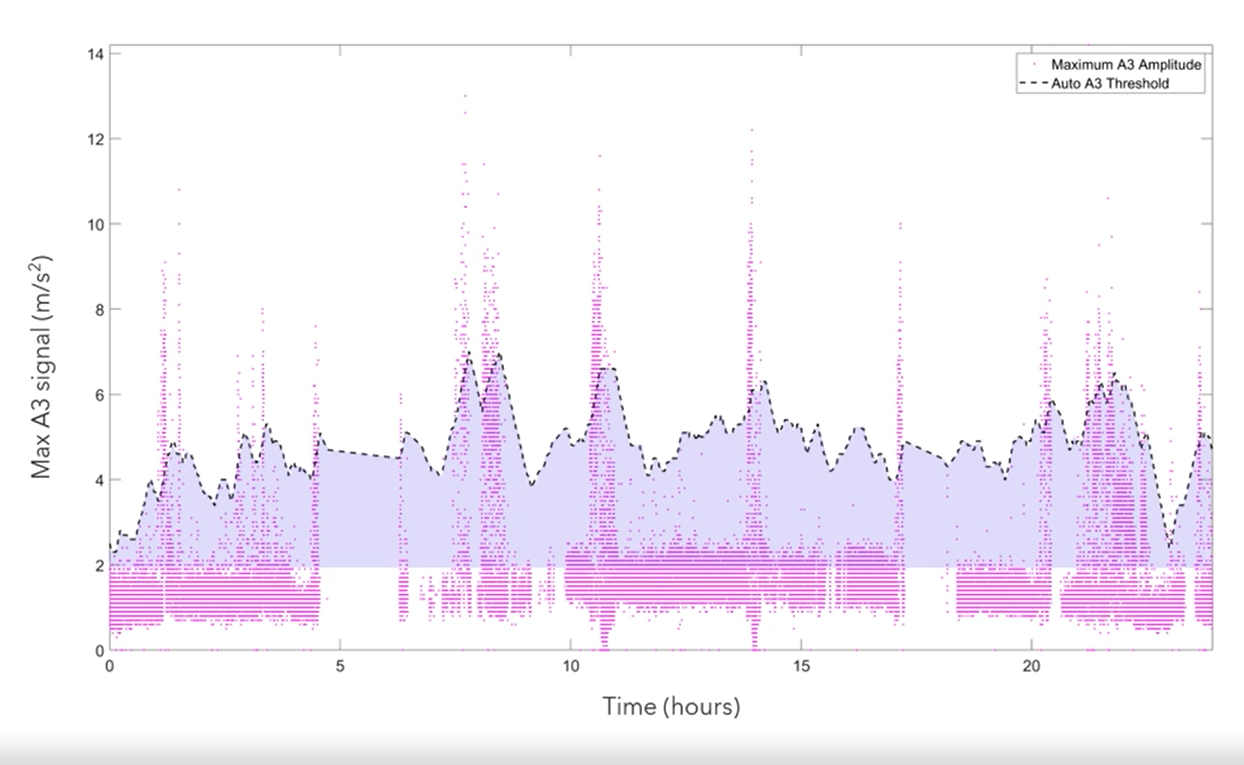


The maximal A3 signal amplitude is stored for 24 hours. The dashed line represents the A3 threshold set by the Auto A3 Threshold algorithm. There is undersensing of the A3 signal represented by the shaded blue. The recommendation is to turn Auto A3 Threshold to “off” and set a fixed A3 threshold.

**Supplementary Figure 2. Post Ventricular Atrial Blanking.**


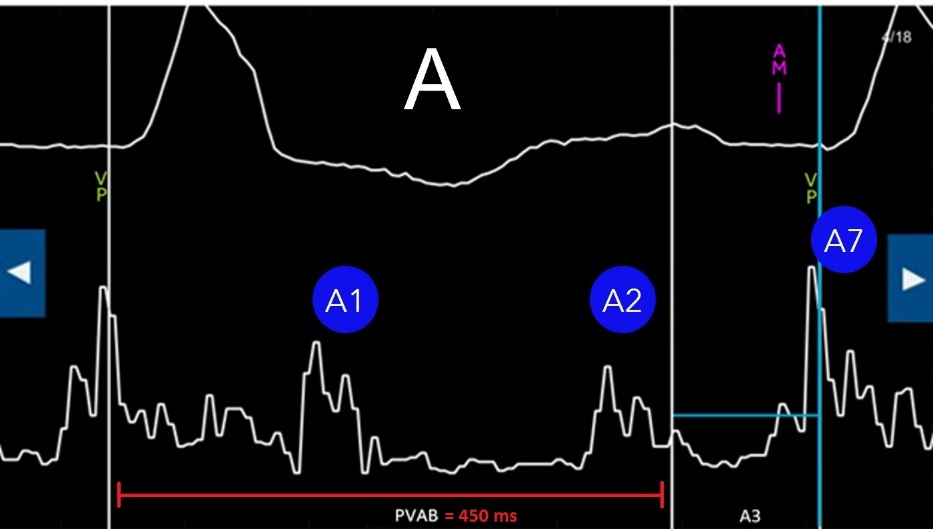

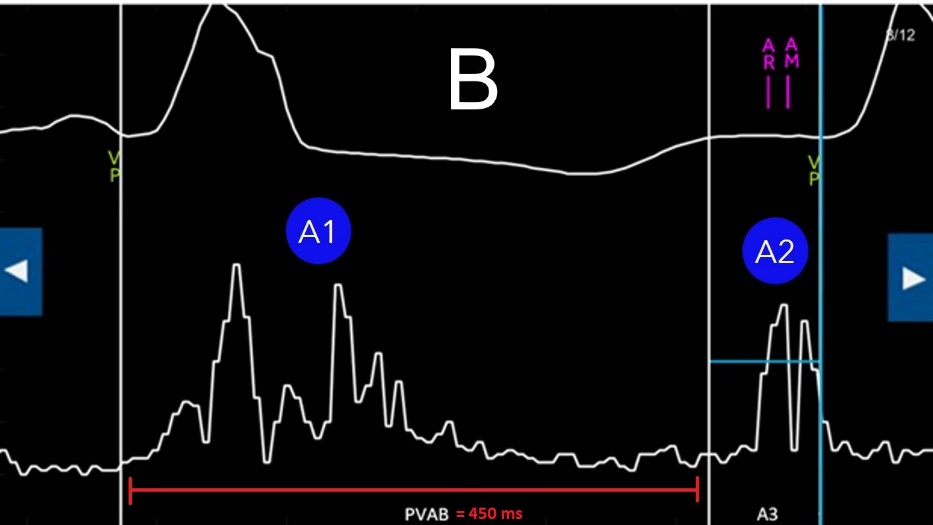


These Figures shows two cycles with a fixed PVAB programming. The top graph (A) shows the accelerometer signals at high sinus rate with appropriate tracking of an A7 signal in the A3 window. The bottom graph (B) shows oversensing of the A2 signal. The A2 signal can occur later in the cardiac cycle as sympathetic drive diminishes and heart rate slows. If PVAB is programmed too short, the A2 signal can be oversensed and lead to inappropriate tracking of the A2 signal. The AR marker denotes the first detection of the A2 occurring within the PVARP interval.
